# Counting the Invisible: New Tools to Estimate the Number of Contributors From Sequence‐Based Microsatellite Genotyping of Environmental DNA Samples

**DOI:** 10.1111/1755-0998.70051

**Published:** 2025-09-26

**Authors:** Olivier Lepais, Ivan Paz‐Vinas

**Affiliations:** ^1^ Univ. Bordeaux, INRAE BIOGECO Cestas France; ^2^ Université Claude Bernard Lyon 1 LEHNA UMR 5023, CNRS, ENTPE Villeurbanne France

The study of intraspecific genetic variation in environmental DNA samples has recently gained momentum following its demonstration as an effective method to study population‐level processes (Andres et al. 2023). Although allele frequencies can be inferred from the distribution of allele sequence coverage within a sample, the number of detected alleles can be used to estimate the number of contributors (NOC), a long‐standing issue in forensic science. This development enables the estimation of the absolute abundance of a species, opening up new possibilities for population monitoring and ecological and evolutionary studies. Although promising, no dedicated tools providing a straightforward way to implement it existed. In this issue of *Molecular Ecology Resources*, Liggan et al. (2025) make a welcome contribution to the field by introducing two new R packages that facilitate the estimation of multi‐locus allelic diversity and of the NOC from the sequencing of microsatellites of mixed samples obtained from environmental DNA. The amplicomsat R package determines the observed allele count (based on sequence length and sequence identity) from sequence‐based microsatellite genotyping (Figure 1A). The genotypequant R package estimates the NOC given the number of observed alleles within mixed samples and allele frequencies of a reference population (Figure 1B). The authors conducted extensive testing of the developed method using simulation and empirical work in the laboratory and in the field, providing a convincing demonstration of its strengths and limitations, but also useful guidelines for future applications to other biological models or to address a broad range of scientific inquiries. Importantly, these advances can help support ongoing global biodiversity monitoring efforts.

**FIGURE 1:**
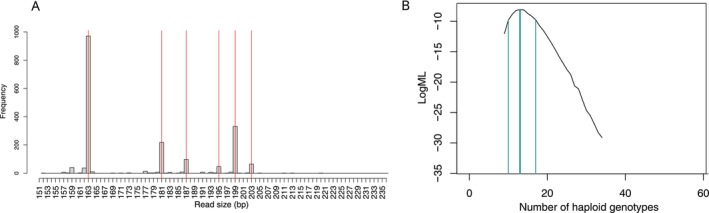
Microsatellite microhaplotype scoring from amplicon sequencing of individuals and environmental DNA in amplicomsat (A) and estimation of the number of contributors (haploid genotypes) in genotypequant (B). Note that if the output of amplicomsat can be directly used as input genotypequant, each package has its own purpose and can be used independently. In particular, genotypequant necessitates simple input data that can be generated with a wide range of upstream processing tools. This is a welcome feature that should encourage creative use of the method which may have unforeseen applications, such as for instance inferring individual ploidy in species with variable ploidy level. Courtesy of Lauran Liggan.

The results reported by Liggan et al. (2025) are promising because the method performed well with easy‐to‐gather molecular data. Using 11 microsatellites and 40 individuals as references, it revealed a total of 177 alleles differing by their size or 297 microhaplotypes based on allele sequence identity (averaging 16 and 27 alleles per locus, respectively), with satisfactory accuracy with up to 20 contributors (Liggan et al. 2025). This was the case for the bull kelp gametophyte study (Figure 2), where empirical validation was conducted on eight contributors, and field sample estimates ranged from one to 12 contributors. However, more complex mixtures (> 50 contributors) as well as missing genotypes due to degraded DNA in environmental samples were more challenging, calling for caution when applying the method in these specific cases. Improving PCR efficiency or library preparation can help generate more data from difficult samples, as suggested by the authors. It is worth noting; however, that Liggan et al. (2025) were able to estimate the NOC from field samples with reasonable confidence even in the presence of high sequencing failure (50% of missing genotypes among field samples). This illustrates how the method can be successfully applied in a realistic scenario, adding up to previous case studies that also made significant advances in the field (Andres et al. 2021). In the author's study case, the additional information provided by allele sequence identity (compared to allele size) was crucial to recover the expected correlation between the NOC and the surface area sampled. This empirical result illustrates the wealth of information provided by considering all polymorphisms in the sequenced amplicon encoded as a microhaplotype. In a recent application targeting 74 nuclear loci using GT‐seq with bioinformatics analysing SNPs, (Shi et al. 2025) identified 252 unique microhaplotypes among 565 Chinook salmon (mean 3.4 alleles per locus). This curated dataset provides enough power to resolve a mixture of up to 10 individuals, with minimal headroom to accommodate high missing genotypes on degraded DNA from digestive tracts. The number of observed alleles is especially important for accurate inference. Thus, integrating highly polymorphic variants such as microsatellites is ideal to maximise the number of alleles within a short DNA fragment. Such compact markers are also more likely to amplify during PCR in the context of degraded environmental DNA. It is important to note that Liggan et al. (2025) sampled specific microhabitats where their studied organism was known to be present, increasing the probability of capturing DNA from the target species. Applying the approach to sampling highly diluted DNA dispersed in the environment could prove to be more challenging.

**FIGURE 2:**
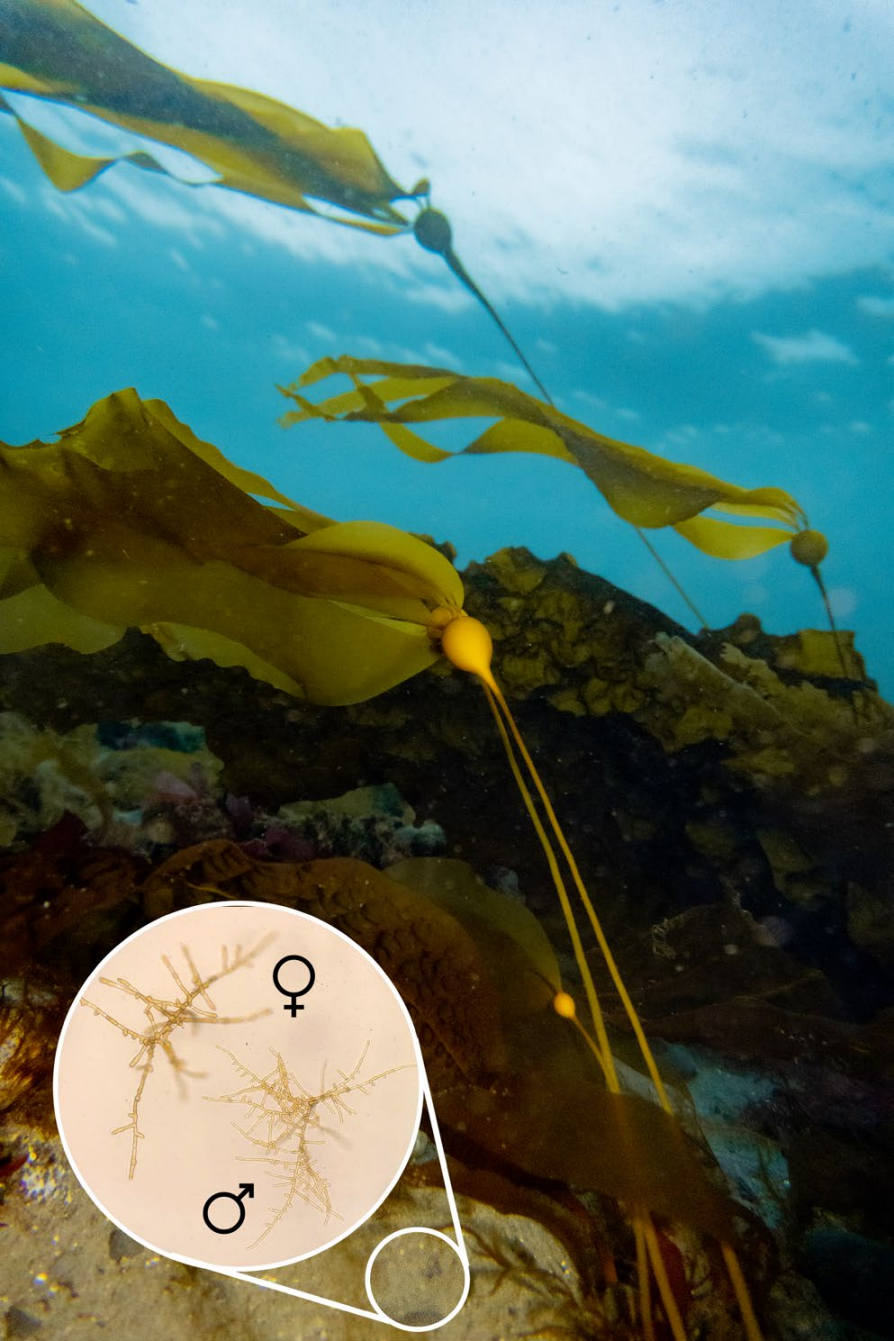
Picture of sporophytes and microscopic gametophytes (insert) of bull kelp. Bull kelp (*Nereocystis luetkeana*) exhibits a complex life‐history, in which microscopic haploid gametophytes (insert: Microscopy image by Gabriel Montecinos), residing on the benthos, alternate with macroscopic diploid sporophytes (captured in Campbell River, British Columbia, Canada, by Bartek Radziej). The sporophyte stage forms extensive and structurally complex underwater forest ecosystems. If sporophyte abundance can be observed in the field, the population size of cryptic gametophytes remained unknown until the advent of genetic diversity analysis of environmental DNA. The tools developed by Liggan et al. (2025) make the estimation of the number of contributors from a DNA mixture easier than ever. Courtesy of Lauran Liggan, Bartek Radziej and Gabriel Montecinos.

Simulations and the empirical validation conducted by Liggan et al. (2025) clearly indicate room for improvement in cases involving more complex DNA mixtures or less polymorphic markers, including by increasing the number of markers, the number of individuals in the reference sample or the number of detected alleles. The latter solution can involve sequencing longer markers, which will increase the cumulated number of detected polymorphisms and hence the total number of observed alleles, as illustrated in humans with hundreds of macrohaplotypes predicted for 8 kb markers spanning multiple microsatellites (Ge et al. 2021). As the number of observable alleles increases, additional power is available to resolve complex DNA mixtures without suffering from locus saturation (Andres et al. 2023). The genotypequant R package thus represents a welcome improvement over previous implementations, as it tolerates high numbers of alleles while offering greater computational efficiency for handling complex mixtures and high polymorphism.

Another way for progress is reducing genotyping error to detect rare variants (Andres et al. 2021), which could be achieved through the use of the emergent digital sequencing (Andersson et al. 2024). By tagging each original DNA molecule with a unique molecular index in early protocol steps, digital sequencing allows determining a consensus sequence of each original DNA strand and greatly improves the distinction between rare variants and errors introduced during PCR and sequencing (Carlson et al. 2015). Although there are many ways to implement digital sequencing, only a few are probably appropriate for degraded and weakly concentrated eDNA, which calls for further specific testing and development. Hybrid‐capture sequencing, which has been successfully used (Ai et al. 2025) to estimate the abundance of two goby species by focusing on substitutions in enriched mitochondrial DNA, might be more efficient than PCR‐based approaches for degraded DNA.

Even without further technical improvements, microhaplotype data are now readily accessible for processing in the tools developed by Liggan et al. (2025) to estimate the number of individuals releasing gametes in the environment during mating. It opens up new possibilities to understand complex ecological processes, such as, for instance, plant pollination through the study of pollen transported by insects (Kämper et al. 2025) or of wind‐dispersed pollen captured by airborne passive samplers (Lin et al. 2025). This new kind of data will provide new information about the plant reproductive landscape. As pointed out by the authors, their method enhances the capabilities of eDNA to monitor further beyond presence‐absence populations of species with complex life histories, or that are microscopical, elusive or rare enough to be monitored using traditional direct sampling methods.

Besides, the advances made by Liggan et al. (2025) are important to support global biodiversity monitoring efforts. Methods that would simultaneously determine both community‐level species diversity and intraspecific diversity across multiple species remain panaceas for evolutionary and conservation biologists. Although numerous challenges persist, such as accurately estimating exact allele frequencies or summary statistics, like heterozygosities from eDNA samples, the methodological advances made by Liggan et al. (2025) and others (Andres et al. 2021) are paving the way towards this goal.

As allelic variation is a key feature in evolutionary biology and conservation (Allendorf et al. 2024), observed allele counts obtained from species‐specific microsatellites sequenced from environmental samples provide highly valuable information from a conservation standpoint. First, observed allele counts can serve as proxies for allelic richness, that is, one of the six essential biodiversity variables for monitoring genetic composition (Hoban et al. 2022). Furthermore, observed allele counts may reveal rare or private alleles in specific populations, informing about their genetic uniqueness (Kalinowski 2004). Finally, multi‐specific observed allele counts can be incorporated into systematic conservation planning tools to identify priority areas for intraspecific genetic diversity conservation (Paz‐Vinas et al. 2018).

The NOC and the derived individual densities could help approximate population census sizes (Nc), a key metric for population monitoring. These estimates can help compute the headline genetic indicator A.4 of the Kunming‐Montreal Global Biodiversity Framework of the UN's Convention on Biological Diversity (i.e., the proportion of populations within species with an effective population size Ne above 500), given the links existing between Ne and Nc (Allendorf et al. 2024; Mastretta‐Yanes et al. 2024). As countries are increasingly deploying eDNA‐based biomonitoring programmes, integrating procedures like those developed by Liggan et al. (2025) could help optimise these approaches, unlocking the possibility to perform multifaceted biodiversity monitoring—from genes to communities—in cost‐effective ways.

## Conflicts of Interest

The authors declare no conflicts of interest.

## Data Availability

No new data produced for this article.
